# Preparation of Low Volatile Organic Compounds Silver Paste Containing Ternary Conductive Fillers and Optimization of Their Performances

**DOI:** 10.3390/molecules27228030

**Published:** 2022-11-19

**Authors:** Youliang Cheng, Jin Zhang, Changqing Fang, Wenke Qiu, Hao Chen, Haonan Liu, Ying Wei

**Affiliations:** Faculty of Printing, Packaging Engineering and Digital Media Technology, Xi’an University of Technology, Xi’an 710048, China

**Keywords:** conductive silver paste, Ag microflakes, Ag nanoparticles, Ag nanowires, ternary fillers

## Abstract

Conductive silver paste is a key material in the fields of printed circuits and printed electronic devices. However, the preparation of conductive silver paste with low-cost and volatile organic compounds (VOCs) is still a challenge. In this work, conductive silver pastes with excellent comprehensive performances were developed by using water-borne polyurethane (WPU) as the bonding phase and using the ternary mixture of Ag microflakes (Ag MFs), Ag nanowires (Ag NWs), and Ag nanoparticles (Ag NPs) as the conductive phase. WPU endowed conductive silver pastes with the adhesion along with releasing a few VOCs during the curing. Results showed that a small amount of Ag NPs or Ag NWs dramatically enhanced the electrical conductivity of silver paste paint film filled only with Ag MFs. The electrical resistivity for optimal ternary mixture conductive silver paste was 0.2 × 10^−3^ Ω∙cm, and the conductive phase was composed of 20.0 wt% Ag MFs, 7.5 wt% Ag NWs, and 2.5 wt% Ag NPs. Meanwhile, the adhesive strength and hardness of silver paste paint film were effectively improved by increasing the curing temperature. The optimal overall performance of the conductive silver pastes was achieved at the curing temperature of 160 °C. Therefore, this work can provide a new route for preparing conductive silver pastes with high performances.

## 1. Introduction

As an important material in the electronic industry, conductive silver pastes have excellent application prospects in light-emitting diode (LED), liquid crystal display (LCD), integrated circuit (IC) chips, and other electronic devices due to their outstanding performances [1,2,3,4,5,6]. Conductive silver paste as a functional ink is prepared by mixing the conductive phase, bonding phase, solvent, and other additives. The bonding phase will provide the basic mechanical properties while the conductive phase provides the conductive pathway [7]. Common oil-soluble resin binders such as epoxy resins [8], phenolic resins [9], and polyamide resins [10] easily release volatile organic compounds (VOCs) during drying and curing. To reduce VOCs, waterborne polyurethane (WPU) is chosen as the binder [5,9,11]. In addition, the conductive phase is mainly composed of the metals Au, Ag, Cu, Al and Ni [12,13]. Currently, Ag has become the best conductive phase because it has the lowest resistivity [14]. Ag fillers including particles, flakes, nanowires and so on, are generally used as typical conductive phases, and their morphology and structure have an important influence on the properties of the conductive silver paste [15].

According to previous reports, multi-dimensional conductive fillers could form more effective conductive network and improve electrical conductivity (EC) [16,17,18]. Behnam et al. [19] significantly improved EC by adding Ag NP deposited on the graphene surface to the traditional conductive silver paste composed of silver flakes and epoxy resin, where the average particle size of Ag NP was 9 nm. Zhang et al. [20] reported that the resistivity of conductive nanocomposites with low resistance composed of epoxy resin, Ag micro-flakes (Ag MFs), and Ag NPs was only 8.1 × 10^−5^ Ω∙cm. For the conductive silver paste, mutual doping of Ag MFs and Ag NPs with small sizes can better fill the gaps between Ag MFs and they form more conductive pathways [21]. Interestingly, Ag nanowires (Ag NWs) also show excellent electron conduction bridges due to the special morphology and a high aspect ratio in addition to the nano-size effect [22,23,24].

To obtain low-temperature curing silver pastes with high conductivity, the filling amount of single Ag fillers was up to 60–90 wt%, which led to a reduction in their bonding properties [25,26,27,28]. Studies have shown that fractal silver dendrites with diverse nano-silver structures and multi-level fractal structures can sinter at 60 °C [29,30,31] and blending Ag fillers can reduce the percolation threshold of silver paste [32]. In addition, WPU can be used as a binder of silver paste because the development of conductive silver paste is currently moving in the direction of a green strategy [33,34]. To our knowledge, this film had good abrasion resistance and minor film shrinkage, corrosion, and chemical resistance after curing [35,36].

In this work, composite conductive silver pastes were prepared by using Ag MFs, Ag NPs, and Ag NWs as conductive fillers and WPU as the binder phase. As the environmentally friendly conductive silver paste, its EC also can be optimized by doping Ag NPs and/or Ag NWs with Ag MFs. Also, the increase in curing temperature effectively improved the adhesive strength and hardness of conductive silver paste. As-prepared conductive silver pastes exhibited good practicability when maintaining conductivity.

## 2. Results and Discussion

### 2.1. Morphology and Microstructure of Ag NPs, Ag NWs and Ag MFs

**Figure 1:**
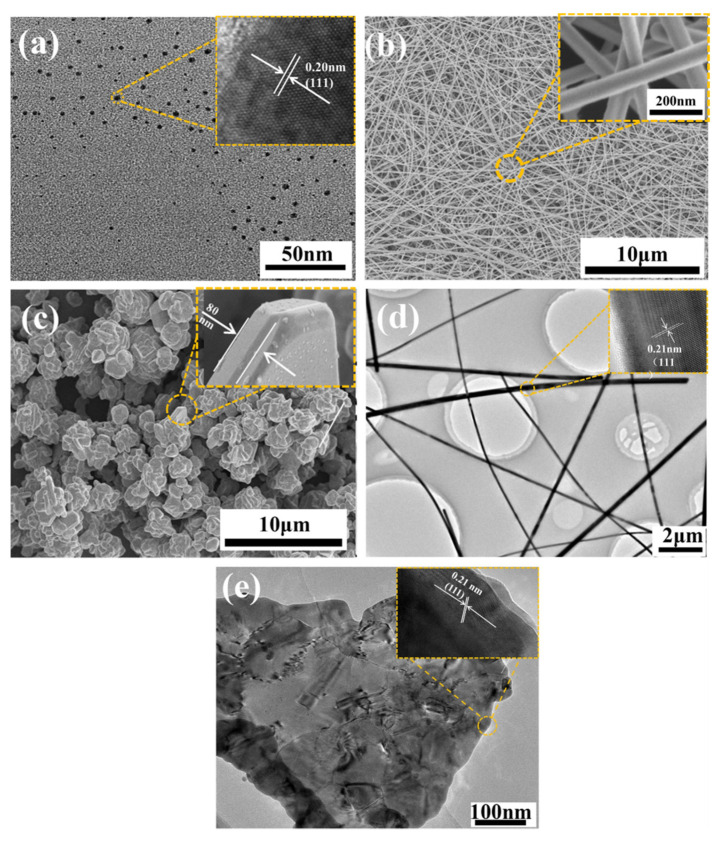
TEM image of (**a**) Ag NPs, SEM images of (**b**) Ag NWs and (**c**) Ag MFs, TEM images of (**d**) Ag NWs, and (**e**) Ag MFs.

**Figure 2:**
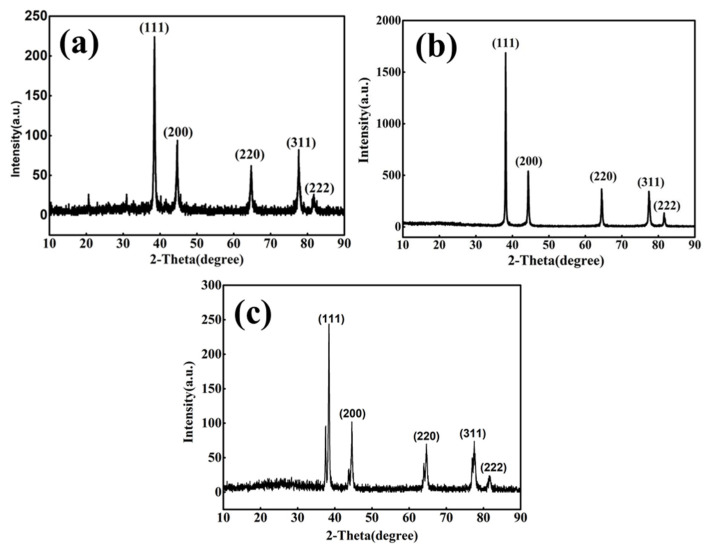
XRD patterns of (**a**) Ag NPs, (**b**) Ag NWs, and (**c**) Ag MFs.

The TEM image of the Ag NPs (Figure 1a) shows that the Ag NPs were well-distributed throughout the sample without obvious agglomerations. Particle size distribution of Ag NPs was derived from a sequence of TEM images similar to Figure 1a using Nano Measurer Software. The mean particle size of Ag NPs was approximately 3.96 nm (in Figure S2a). SEM images of the Ag NWs and Ag MFs are presented in Figure 1b,c, respectively. The mean length and diameter of Ag NWs were clearly measured to be 10.51 μm and 60.02 nm, respectively (in Figure S2b and Figure S2c). In addition, the mean aspect ratio of Ag NWs was calculated to be approximately 250. The thickness and size of Ag MFs were measured to be about 80 nm and 1.5 μm, respectively (in Figure 1c and Figure S2d). Furthermore, according to TEM images of Ag NWs and Ag MFs, it can be observed that morphologies are similar to SEM images and both grow along the (111) crystal plane with a high crystallinity (in Figure 1d,e). It can be seen from the XRD pattern of Ag NPs (in Figure 2a) that 2θ = 38.48°, 44.7°, 64.74°, 77.64°, and 81.72° peaks were corresponded to (111), (200), (220), (311), and (222) crystal planes of Ag, respectively. It matched well the peaks of the XRD standard card of pdf#87-0717, which also were observed in XRD patterns of Ag NWs and Ag MFs (in Figure 2b,c). Thus, XRD results with few miscellaneous peaks indicate that the purity and crystallinity of Ag NPs, Ag NWs, and Ag MFs prepared in advance are high.

### 2.2. Performances of Paint Film Filled Only with Ag MFs

**Figure 3:**
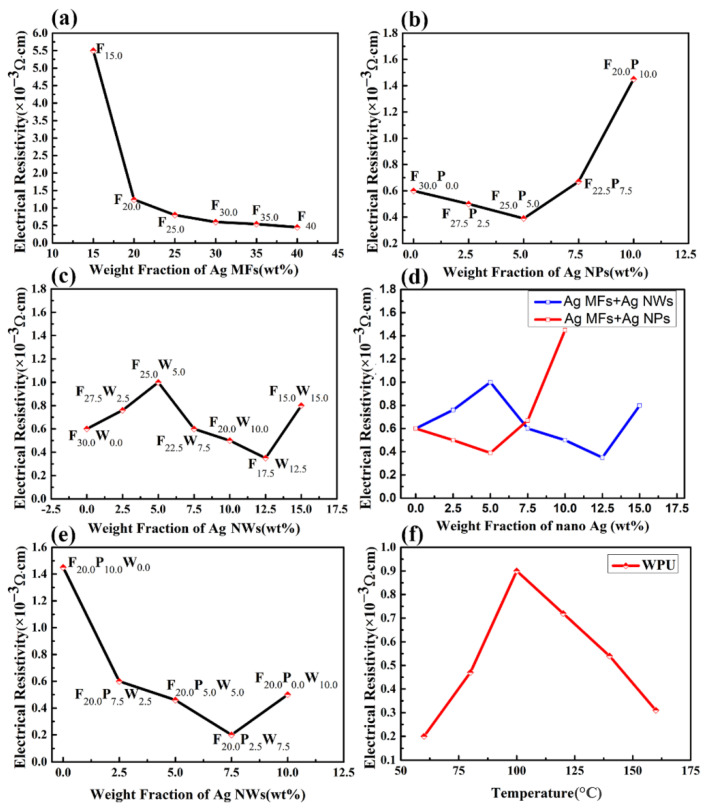
(**a**) ER of the conductive silver paste filled only with Ag MFs after curing at 60 °C, (**b**) ER of the binary mixture conductive silver paste containing Ag MFs and Ag NPs after curing at 60 °C, (**c**) ER of the binary mixture conductive silver paste containing Ag MFs and Ag NWs after curing at 60 °C, (**d**) ER comparison of two binary mixture conductive silver pastes after curing at 60 °C, (**e**) ER of ternary mixture conductive silver paste containing Ag MFs, Ag NPs and Ag NWs after curing at 60 °C, and (**f**) ER of the sample F_20.0_P_2.5_W_7.5_ using WPU as the binder at different curing temperatures.

**Figure 4:**
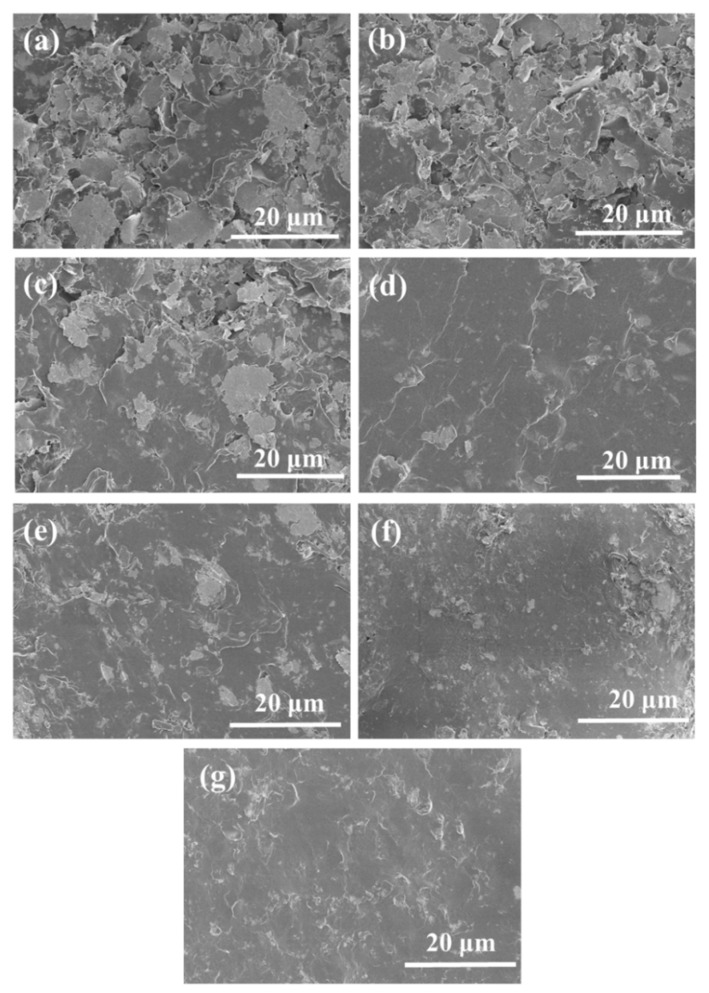
SEM images of the cross sections of paint film single containing different amounts of Ag MFs after curing at 60 °C: (**a**) F_10_, (**b**) F_15_, (**c**) F_20_, (**d**) F_25_, (**E**) F_30_, (**f**) F_35_ and (**g**) F_40_.

According to Figure 3a, the electrical resistivity (ER) of paint film containing only Ag MFs increased with the increasing amount of Ag MFs. As shown in Figure 4a, many gaps can be observed between Ag MFs in the paint film filled only with Ag MFs. When the loading level of Ag MFs gradually increased, close sheets (in Figure 4b–g) resulted in the decrease of tunneling resistance (R_t_) [15]. Then, the conductivity (C) of these samples increased from 181.8 S/cm to 2500 S/cm. Moreover, it was observed that the conductivity of the sample with 30 wt% Ag MFs was very close to that with 35 wt% Ag MFs, and subsequent C change was weak.

According to Table 1 and Figure S3, we find that the adhesive strength and a pencil hardness of the paint films filled only with Ag MFs decreased with increasing the amount of Ag MFs. The adhesion and hardness of the paint films changed to be poor when the amount of Ag MFs fillers was high (>30 wt%). Thus, the adhesive strength grade of sample F_30_ was grade 2, but the spalling area of samples F_35_ and F_40_ exceeded 15% and changed to grade 3. In particular, the hardness grade of F_35_ and F_40_ was only HB and B, respectively. This result may be ascribed to that accumulated gaps between Ag MFs makes the cured paint film compact insufficiently [26]. Therefore, the total amount of Ag fillers in the conductive silver pastes will be determined as 30 wt% to achieve outstanding overall performances.

### 2.3. Performances of Paint Films Containing Binary Mixture Ag Fillers

**Figure 5:**
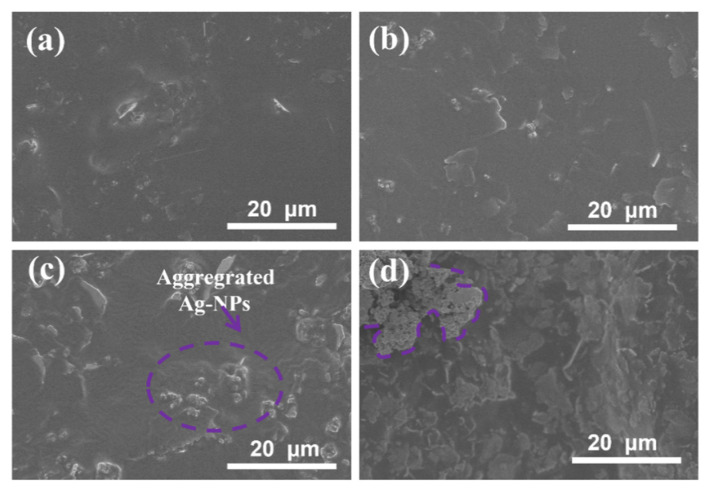
SEM images of silver paste paint films containing Ag MFs and Ag NPs after curing at 60 °C: (**a**) F_27.5_P_2.5_, (**b**) F_25.0_P_5.0_, (**c**) F_22.5_P_7.5_, and (**d**) F_20.0_P_10.0_.

As shown in Figure 3b, the ER of the paint films containing Ag MFs and Ag NPs first decreased with the increase of Ag NPs amount, then increased after more than 5 wt%. Generally, the resistance of the paint film is determined by the interface contact resistance (R_i_) between neighboring fillers [7,15], and the total R_i_ is the sum of the constriction resistance (R_c_) and tunneling resistance (R_t_) [15,26]. An excellent conductive network can be formed when a low amount of Ag NPs was filled in the gaps between Ag MFs [7,19]. In addition, a lot of Ag NPs tend to sinter or Ostwald ripen at low temperatures, generating protrusions as depicted by the purple lines in Figure 5c,d, which can lower the total free energy [37]. Then, the R_t_ of paint films containing Ag MFs and Ag NPs cannot reduce much due to these aggregated Ag NPs [7,15]. Therefore, the R_i_ between neighboring regions containing Ag NPs rose rapidly as the number of Ag NPs increased further to a high level [15,19].

The adhesive strength grade of the binary mixture paint films can be improved when the amount of Ag NPs was less than 7.5 wt%. The edge of the sample F_27.5_P_2.5_ and F_25.0_P_5.0_ notch was flat in the groove and then the edge of the lattice was free of any peeling with the adhesive strength grade from 2 to 0 according to Table 2 and Figure S4I(a–d). This result may be due to a larger specific surface area and a higher surface energy of Ag NPs than Ag MFs. In addition, Ag NPs are easier to be wet by WPU [25]. In contrast to the changes in adhesive strength grade, the hardness grade of the paint films increased to 2H when the amount of Ag NPs was up to 10 wt% (in Figure S4II(a–d)). The hardness grade is related to the composition of the fillers, and the high amount of Ag NPs will be denser which has an obvious positive effect on the hardness improvement. Therefore, the proper addition of Ag NPs can improve pencil hardness and adhesive strength.

**Figure 6:**
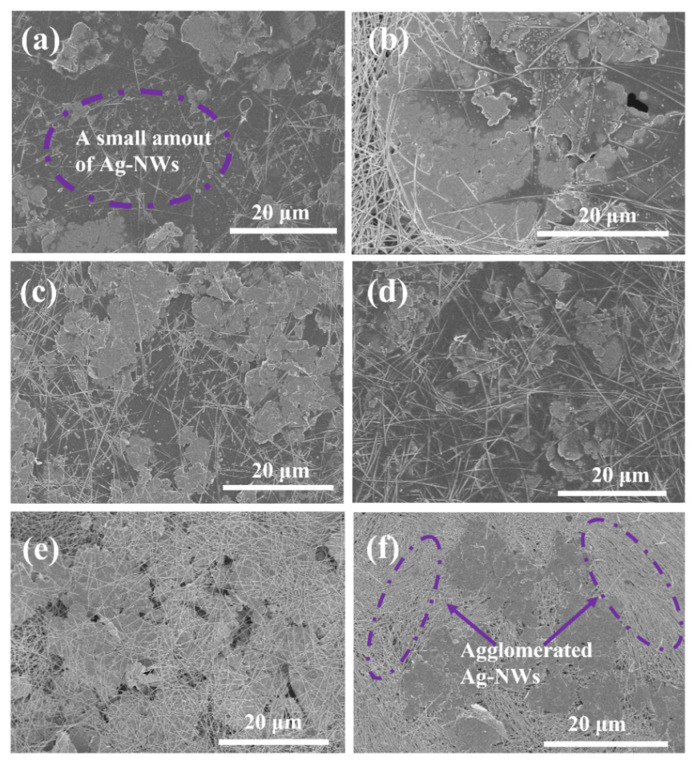
SEM images of silver paste paint films containing different amounts of Ag MFs and Ag NWs after curing at 60 °C: (**a**) F_27.5_W_2.5_, (**b**) F_25.0_W_5.0_, (**c**) F_22.5_W_7.5_, (**d**) F_20.0_W_10.0_, (**e**) F_17.5_W_12.5_, (**f**) F_15.0_W_15.0_.

As shown in Figure 3c, the ER of the paint films containing Ag MFs and Ag NWs first increased with the Ag NWs amount increasing to 5.0 wt% and then decreased with further increases of Ag NWs amount until 12.5 wt%. However, ER had a sharp increase when the amount of Ag NWs was up to 15 wt%. The average diameter of Ag NWs is much lower than the thickness of Ag MFs according to the morphology analysis. The Ag NWs cannot act as a lap between Ag MFs with large voids when a small number of Ag NWs (<5.0 wt%) was mixed with Ag MFs (in Figure 6a). It can act as a lap until the amount of Ag NWs reached 7.5 wt%. Then, more electrically conductive channels were formed (in Figure 6b–e), leading to a sharp decrease in tunneling resistance [15,29]. Besides, the constriction resistance slightly increased due to the addition of a certain amount of Ag NWs. Thus, the interface contact resistance decreased due to the above two reasons. As a result, the ER of the paint film decreased when the amount of Ag NWs exceeded 5 wt%. However, a very high amount of Ag NWs with uneven dispersion will destroy the original conductive channels of Ag MFs. At the same time, the mutual entanglement of Ag NWs will promote the increase of contact points and the tunnel resistance indicated by purple dotted lines in Figure 6f [1,15,21]. Therefore, the ER of the paint film increased as the amount of Ag NWs was over 12.5 wt%.

The adhesive strength and pencil hardness grade of the binary mixture paint films decreased initially with the increase of the amount of Ag NWs (in Figure S5a–f). It can be seen from Table 3 that the hardness of the paint film decreased to HB when the amount of Ag NWs was 7.5 wt%. This result is due to be that a high amount of Ag NWs with more residual solvent has a negative impact on the hardness of the paint film. Compared with Ag NPs, it is difficult to wet Ag NWs by WPU due to the smaller specific surface area of Ag NWs. Therefore, the grade of pencil hardness and adhesive strength of binary mixture paint films were reduced by the addition of Ag NWs.

A comparison of ER between different binary mixture paint films is shown in Figure 3d. It can be concluded that an appropriate amount of both Ag NPs and Ag NWs can improve the EC of the conductive silver pastes containing only Ag MFs. Among them, when the amount of Ag MFs was 20 wt%, the optimized amount for only Ag NPs and Ag NWs was 5 wt% and 10 wt%, respectively. Furthermore, the effect of adding Ag NWs on reducing the ER of the paint film was more obvious compared with Ag NPs, as the former relied on a large aspect ratio and formed conductive networks more easily. For the hardness and adhesive strength, Ag NPs showed a positive effect on the silver paste while the amount of Ag NWs should not exceed 10 wt%. Besides the performances, considering the cost of silver paste, the total amount of Ag fillers was determined to be 30 wt% where the amount of Ag MFs was 20 wt% and the total amount of Ag NWs and Ag NPs was 10 wt%. Then, a ternary mixture of conductive silver paste with the optimum conductivity was obtained by optimizing the amount of Ag NWs and Ag NPs. 

### 2.4. Performances of Paint Films Containing Ternary Mixture Ag Fillers

**Figure 7:**
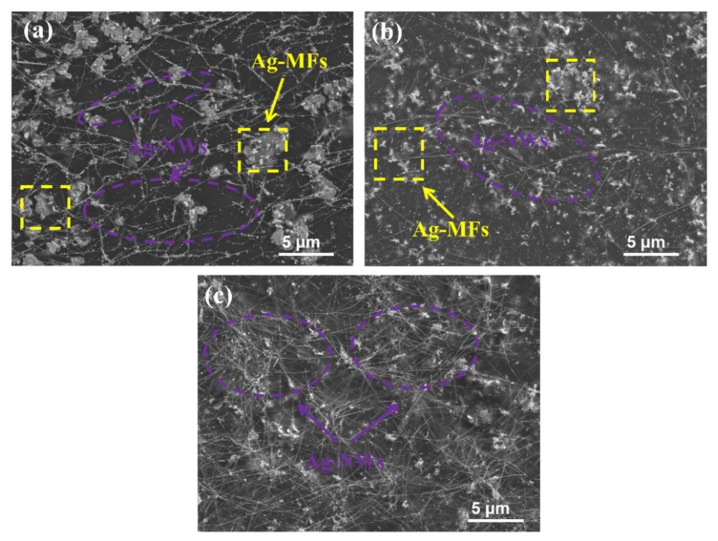
SEM images of silver paste paint films containing different amounts of Ag MFs, Ag NWs, and Ag NPs after curing at 60 °C: (**a**) F_20.0_P_7.5_W_2.5_, (**b**) F_20.0_P_5.0_W_5.0_, and (**c**) F_20.0_P_2.5_W_7.5_.

As shown in Figure 3e, the ER of the ternary mixture paint film decreased as the amount of Ag NWs increased while the amount of Ag NPs decreased. On the one hand, Ag NWs have a high mean aspect ratio (250), which facilitates the establishment of conducting networks [23,24]. Ag NWs acted as a lap between Ag MFs, and Ag NPs can attach to the surface of Ag NWs as shown in Figure 7a. When the number of Ag NWs was over 5 wt%, the conductive network became dense as shown in Figure 7b,c. Due to the greater volume fraction of Ag NWs compared with Ag NPs, mutual contact points of Ag fillers will decrease sharply when the amount of Ag NPs decreased and the amount of Ag NWs increased, resulting in a sharp decrease of interface contact resistance. Therefore, the EC of the paint films containing ternary mixture Ag fillers is relatively good. Among these samples, F_20.0_P_2.5_W_7.5_ exhibited the lowest ER of 0.2 × 10^−3^ Ω∙cm where Ag NWs overlapped with Ag MFs to build the conductive network, and Ag NPs were filled in the small gaps of Ag MFs to effectively supplement this network.

Although the pencil hardness grade of the ternary mixture paint film had no significant change, the adhesive strength gradually decreased with the increase of the amount of Ag NWs according to Table 4. The paint film changed from partial peeling off to large pieces peeling off along the edge of the notch as shown in Figure S6a–c, and the adhesive strength grade changed from grade 1 to grade 2 or even grade 3. This result indicated that the increase of Ag fillers type could reduce the wettability between Ag fillers and WPU. Although the ternary mixture Ag fillers can increase the conductivity, it will have an adverse effect on the adhesive strength of the paint film. 

In general, the dryness process has an impact on the pencil hardness and adhesive strength of paint film. The pencil hardness of the ternary mixture paint film was improved as the curing temperature rose (in Table 5 and Figure S7a–f). The removal of the solvent from the paint film caused the hardness of the paint film to change from H to 3H grade at the curing temperature of 140 °C. In addition, less organic solvent reduced the sintering hindrance of Ag MFs, Ag NWs, and Ag NPs, which improved the crystallinity of Ag fillers [20,23]. According to Table 6 and Figure S8a–f, the adhesive strength of the ternary mixture paint film also was improved as the curing temperature increased. When the curing temperature was higher than 100 °C, only small flakes peeled off at the edge of the cut or the intersection of the cut, and the adhesive strength grade of the paint film changed to grade 1. WPU in conductive silver paste gradually formed cross-linking network at a high curing temperature, and then the distance became narrow between resin carrier/Ag fillers and glass substrate. As a result, the curing temperature can effectively improve the pencil hardness and adhesive strength of ternary mixture paint films.

**Figure 8:**
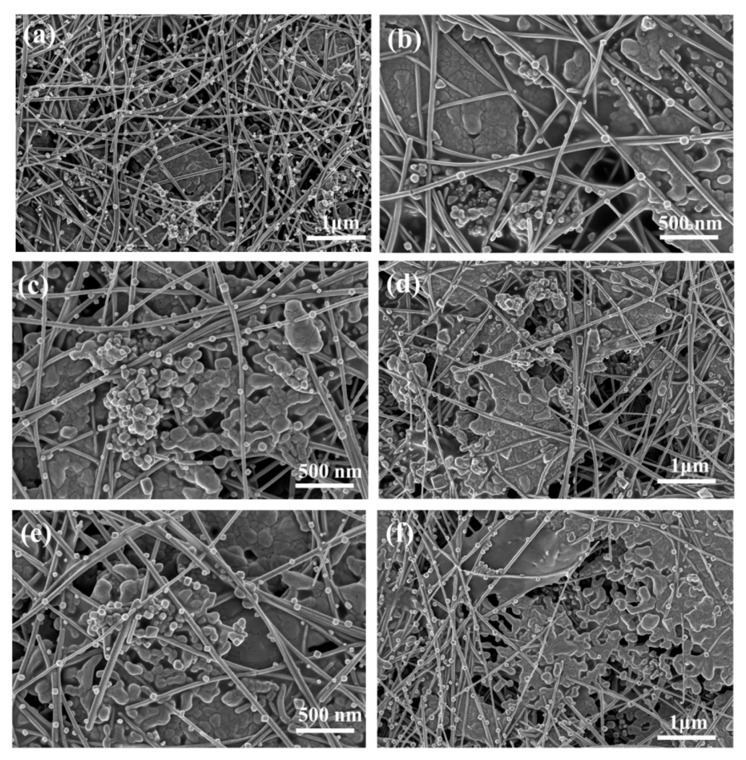
SEM images of silver paste paint film using WPU as the binder (sample F_20.0_P_2.5_W_7.5_) at different curing temperatures: (**a**) 60 °C, (**b**) 80 °C, (**c**) 100 °C, (**d**) 120 °C, and (**e**) 140 °C, (**f**) 160 °C.

**Figure 9:**
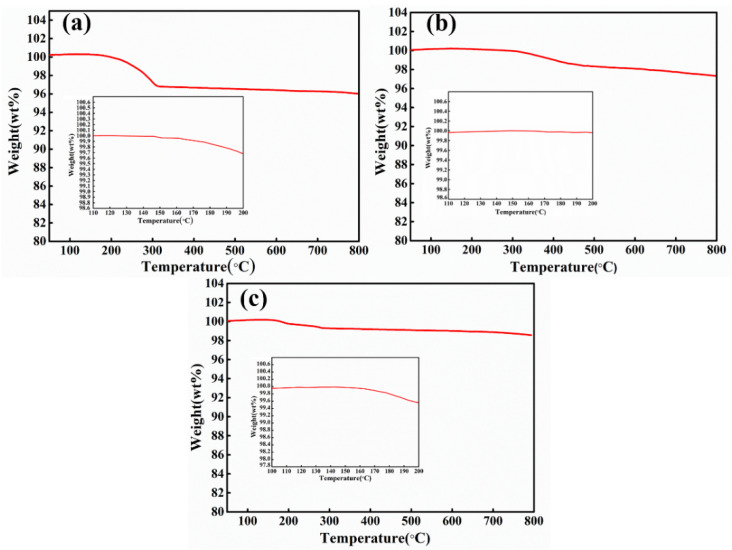
TG curves of (**a**) Ag NPs without other heat treatment, (**b**) Ag NWs without other heat treatment and (**c**) Ag MFs without other heat treatment. The insets show a magnified TG curve in the range of 110–200 °C for Ag NPs, in the range of 110–200 °C for Ag NWs, and in the range of 100–200 °C for Ag MFs, respectively.

The sample with the highest conductivity (sample F_20.0_P_2.5_W_7.5_) was chosen in consideration of the EC performance, pencil hardness, and adhesive strength for investigating the effect of curing temperature on the performances of conductive silver pastes. The ER of the paint film containing 20 wt% Ag MFs, 2.5 wt% Ag NPs, and 7.5 wt% Ag NWs increased initially with the increasing of the curing temperature before 100 °C and then decrease as further elevating the curing temperature as shown in Figure 3f. It can be inferred that the conductive phase in the conductive silver pastes begins to sinter at a certain curing temperature. As shown in Figure 8b,c, Ag MFs had some agglomerates, while both Ag NWs and Ag NPs filled the voids unevenly in the curing temperature range of 80–100 °C, resulting in the increasing of ER for the paint film. Additionally, as shown in Figure 8d,e, Ag NWs, and Ag MFs start to sinter when the temperature exceeds 100°C. These results are consistent with the TG results for Ag NWs and Ag MFs (in Figure 9b,c), both of which already have weight loss at 110°C. The weight of Ag NPs starts to reduce from 110 °C (in Figure 9a), where the organic cladding layer PVP gradually decomposes leading to the loss of potential balance between adjacent Ag NPs as well as the spatial potential resistance effect [38]. Thus, the Ag NPs start to agglomerate and sinter and the ER of the paint film decreases. When the temperature reached 160 °C, Ag NPs exhibited fast weight loss, and a large number of Ag NPs underwent the sintering. At the same time, the sintering of Ag NWs and Ag MFs was also significant (in Figure 8f), thus the ER of the paint film reached the lowest. A comparison of the properties of as-prepared ternary mixture conductive silver pastes in this work and conductive silver pastes in previous reports [1,15,32,39] is shown in Table 7. When the curing temperature was 60 °C, the lowest ER for the ternary mixture paint film with 30 wt% Ag fillers was 0.2 × 10^−3^ Ω∙cm. This ER was comparable to that of previously reported conductive silver pastes with higher content of Ag fillers, but it had a low adhesive strength. At a curing temperature of 160 °C, the best overall performance ternary mixture paint film had an ER of 0.31 × 10^−3^ Ω∙cm and an adhesive strength grade of 1, whereas the conductive silver pastes with equivalent performance in a previous report had Ag fillers content as high as 66.6 wt%. In addition, WPU was used as the carrier in the conductive silver pastes prepared in this work, and then deionized water can be used as the diluent, which will avoid the release of VOCs during the curing process. 

## 3. Materials and Methods

### 3.1. Materials

Silver nitrate (AgNO_3_), ascorbic acid (VC), sodium citrate anhydrous (C_6_H_5_Na_3_O_7_), and hexahydrated ferric chloride (FeCl_3_∙6H_2_O) were supplied by Shengao Chemical Reagent Co., Ltd., Tianjin, China. Poly (N-vinylpyrrolidone) (PVP, MW = ~58,000, ~1,300,000) was obtained from Aladdin reagent Shanghai Co., Ltd., Shanghai, China. Sodium borohydride (NaBH_4_) was supplied by Sinopharm Chemical Reagent Co., Ltd., Shanghai, China, and ethylene glycol (EG) was supplied by Tianjin Baishi Chemical Co., Ltd., Tianjin, China. WPU (WPU-f1526) was supplied by Yoshida Chemical Co., Ltd., Shenzhen, China. The dispersant was an aqueous fluorine-containing surfactant (Capston fs-31) purchased from Kemu Chemical Co., Ltd., Shanghai, China. The coupling agent of γ-(2, 3-epoxypropoxy) propytrimethoxysilane (KH560) was purchased from Shanghai McLean Biochemical Technology Co., Ltd., Shanghai, China.

### 3.2. Preparation of Conductive Phase

Ag NPs were synthesized by the liquid phase reduction method [40,41]. Typically, 10 mL of AgNO_3_ aqueous solution (0.2 M) was added to 10 mL of PVP (MW = ~58,000) ethanol solution (0.2 M), which was mixed and stirred until the color was transparent orange. Then, 10 mL of aqueous solution mixed with NaBH_4_ (0.18 M) and C_6_H_5_Na_3_O_7_ (0.05 M) as the reductant was added to the mixed solution containing AgNO_3_ and PVP. Finally, Ag NPs solution was obtained after 3 h of stirring. 

Ag NWs were synthesized by the solvothermal method [42,43]. Typically, a 40 mL EG solution containing 147 μM FeCl_3_∙6H_2_O was prepared, and then 1.36 g of PVP (MW = ~1,300,000) was added with stirring. Subsequently, a 40 mL EG solution containing 0.1 M AgNO_3_ was prepared. Finally, the above two solutions were transferred into a polytetrafluoroethylene reactor, and the system was heated at 160 °C for 3 h to obtain an Ag NWs solution.

Ag MFs were prepared by hydrothermal method [44,45,46]. Typically, 20 mL of deionized water was fully stirred with 0.544 g of AgNO_3_ and 0.53 g of PVP (MW = ~58,000) (0.53 g). Then, 20 mL of deionized water was stirred with 0.55 mg of FeCl_3_∙6H_2_O and 0.35 g of VC. Subsequently, the mixture solution was put into a 50 mL reactor after being stirred at room temperature for 30 min. The Ag MFs solution was obtained after the system was maintained at 140 °C for 2 h.

After three nano Ag solutions were cooled to room temperature naturally, the solutions were purified by centrifugation. Both Ag NPs and Ag MFs solutions were first washed with ethanol and then with deionized water to separate them from ethanol. Then, the solid Ag NPs and Ag MFs were obtained in a vacuum oven, and they were stored in a brown reagent bottle at a low temperature. The Ag NWs solution was washed with acetone and then ethanol. Lastly, Ag NWs were separated from the ethanol solution using deionized water and stored at low temperatures in a brown reagent bottle. 

### 3.3. Preparation of Composite Conductive Silver Pastes

Firstly, the uniform resin matrix solution was prepared by WPU, deionized water, Capston fs-31, and KH560 in a mass ratio of 100:160:10:10. Secondly, a certain amount of Ag NWs solution was mixed with the above solution. Finally, a certain amount of Ag MFs and Ag NPs were stirred into the above system to obtain conductive silver pastes. The amount of Ag MFs, Ag NWs, and Ag NPs is shown in Table 8. 

### 3.4. Characterization and Measurements

Scanning electron microscopy (SEM, Hitachi S-4300) was used to observe the morphology of Ag MFs, Ag NWs, and conductive silver paste. Transmission electron microscopy (TEM, FEI Tecnai G2 F20 S-Twin) was conducted to observe the morphology of Ag NPs, Ag MFs, and Ag NWs. The purity of Ag was assessed by X-ray diffraction patterns obtained on an X-ray diffractometer (XRD, dx-2500) with CuK_α_ radiation (λ = 0.154 nm) in the range of 10–90°.

The conductivity test method of conductive silver pastes was as follows. Firstly, two parallel lines of 25 mm polyimide tape were placed at a distance of 10 mm along the length of a standard glass slide of 25.4 mm × 76.2 mm. Secondly, the configured silver paste was used to coat the space between the tapes. All the samples were kept in the vacuum oven at a low temperature of 60 °C for 3 h, which helped to observe the dotted line lap phenomenon between different shapes of Ag fillers. The 25 mm tapes were then removed, and the coating thickness was maintained at approximately 50 μm. The schematic illustration of conductive silver paste coating is shown in Figure S1a. The surface resistivity and conductivity of the conductive paint film were detected by a four-probe tester (RTS-9).

The adhesive strength was measured by the Baige knife tester (QFH-A) according to GB/T9286-88. Pencil hardness was measured by a hardness tester (JY-1086) according to GB/T6739-2006. The two Instruments were from Aipu Measuring Instrument Co., Ltd., Quzhou, China. The evaluation standards are shown in Table S1. The schematic for preparing the sample is shown in Figure S1.

Weight losses of the Ag NPs, Ag NWs, and Ag MFs during heating in the N_2_ atmosphere were studied using a thermogravimetric analyzer (TGA, model 209 F3) at a heating rate of 20 °C/min.

A list of the acronyms appearing in this text has been included in Table S2 of the supporting material.

## 4. Conclusions

In summary, ternary mixture Ag fillers/WPU conductive silver pastes with excellent comprehensive performances were made by adding an appropriate amount of Ag MFs, Ag NPs, and Ag NWs to the WPU carrier. The addition of appropriate amounts of Ag NPs and/or Ag NWs can significantly enhance the EC of the conductive silver paste paint film filled only with Ag MFs. The composite conductive silver pastes with the optimal amount of Ag NPs of 2.5 wt%, Ag NWs of 7.5 wt%, and Ag MFs of 20 wt% showed a low ER of 0.2 × 10^−3^ Ω∙cm when curing at 60 °C, but the hardness and adhesive strength of the paint film were poor. Meanwhile, increasing the curing temperature can effectively improve the adhesive strength and hardness of conductive silver pastes paint film. The silver paste paint film had good hardness and adhesive strength while still keeping high EC when the curing temperature was 160 °C. In addition to avoiding the release of VOCs, as-prepared conductive silver pastes with low amounts of Ag in this work showed a low ER when cured at 60 °C while exhibiting the optimal comprehensive performance when cured at 160 °C, which can replace normal conductive silver pastes with high amount of Ag and nonaqueous carrier.

## Figures and Tables

**Table 1:** Hardness and adhesive strength of silver paste paint films single containing different amounts of Ag MFs after curing at 60 °C.

Sample	Ag MFs (wt%)	Adhesive Strength Grade	Hardness Grade
F_10_	10	1	H
F_15_	15	1	H
F_20_	20	1	H
F_25_	25	1	H
F_30_	30	2	H
F_35_	35	2	HB
F_40_	40	3	B

**Table 2:** Hardness and adhesive strength of silver paste paint films containing different Ag MFs and Ag NPs amounts after curing at 60 °C.

Sample	Ag MFs (wt%)	Ag NPs (wt%)	Adhesive Strength Grade	Hardness Grade
F_30.0_P_0.0_	30.0	0.0	2	H
F_27.5_P_2.5_	27.5	2.5	0	H
F_25.0_P_5.0_	25.0	5.0	0	H
F_22.5_P_7.5_	22.5	7.5	1	H
F_20.0_P_10_	20.0	10.0	1	2H

**Table 3:** Hardness and adhesive strength of silver paste paint films containing different amounts of Ag MFs and Ag NWs after curing at 60 °C.

Sample	Ag MFs (wt%)	Ag NWs (wt%)	Adhesive Strength Grade	Hardness Grade
F_30.0_W_0.0_	30.0	0.0	1	H
F_27.5_W_2.5_	27.5	2.5	1	H
F_25.0_W_5.0_	25.0	5.0	1	H
F_22.5_W_7.5_	22.5	7.5	1	HB
F_20.0_W_10.0_	20.0	10.0	1	HB
F_17.5_W_12.5_	17.5	12.5	2	HB
F_15.0_W_15.0_	15.0	15.0	2	HB

**Table 4:** Hardness and adhesive strength of silver paste paint films containing different amounts of Ag MFs, Ag NWs, and Ag NPs after curing at 60 °C.

Sample	Ag MFs (wt%)	Ag NWs (wt%)	Ag NPs (wt%)	Adhesive Strength Grade	Hardness Grade
F_20.0_P_10.0_	20	0.0	10.0	1	H
F_20.0_P_7.5_W_2.5_	20	2.5	7.5	2	H
F_20.0_P_5.0_W_5.0_	20	5.0	5.0	2	H
F_20.0_P_2.5_W_7.5_	20	7.5	2.5	3	H
F_20.0_W_10.0_	20	10.0	0.0	1	HB

**Table 5:** Hardness of conductive silver paste paint films containing Ag MFs, Ag NWs, and Ag NPs at different curing temperatures.

Hardness Grade	60 °C	80 °C	100 °C	120 °C	140 °C	160 °C
F_20_P_7.5_W_2.5_	H	HB	HB	H	3 H	3 H
F_20_P_5.0_W_5.0_	H	H	HB	H	3 H	3 H
F_20_P_2.5_W_7.5_	H	H	HB	HB	3 H	3 H

**Table 6:** Adhesive strength of conductive silver paste paint films containing Ag MFs, Ag NWs, and Ag NPs at different curing temperatures.

Adhesive Strength Grade	60 °C	80 °C	100 °C	120 °C	140 °C	160 °C
F_20_P_7.5_W_2.5_	3	2	1	1	1	0
F_20_P_5.0_W_5.0_	3	2	1	1	1	1
F_20_P_2.5_W_7.5_	3	2	2	1	1	1

**Table 7:** Comparison of the ER of as-prepared composite silver pastes in this work with that of silver pastes in previous reports.

Resin	Conductive Phase	Conductive Phase Amounts (wt%)	Curing Temperature (°C)	ER (10^−3^ Ω∙cm).	Adhesive Strength Grade	Reference
Epoxy Resin	flaky silver pellets	60	90	0.237	-	[1]
	spherical silver pellets					
Polyester resin	Ag microflakes	40	150	0.285	-	[15]
	Ag nanowires					
	Ag nanospheres					
Organic carrier (PVP with 1-hexanol)	silver sphere particles	66.6	175	0.3~0.5	1	[32]
	flake silver powder					
Ethoxyline resin	Cu/Ag fillers (molar ratio of Ag to Cu 2:1)	60	150	0.2~0.5	-	[39]
WPU	Ag microflakes	30	60	0.2	3	This work
	Ag nanowires					
	Ag nanospheres					
WPU	Ag microflakes	30	160	0.31	1	This work
	Ag nanowires					
	Ag nanospheres					

**Table 8:** Sample names of conductive silver pastes with different amounts of Ag.

Sample Name	WPU (wt%)	Ag MFs (wt%)	Ag NWs (wt%)	Ag NPs (wt%)
F_10_	90	10	0	0
F_15_	85	15	0	0
F_20_	80	20	0	0
F_25_	75	25	0	0
F_30_	70	30	0	0
F_35_	65	35	0	0
F_40_	60	40	0	0
F_27.5_P_2.5_	70	27.5	0	2.5
F_25.0_P_5.0_	70	25.0	0	5.0
F_22.5_P_7.5_	70	22.5	0	7.5
F_20.0_P_10_	70	20.0	0	10.0
F_27.5_W_2.5_	70	27.5	2.5	0
F_25.0_W_5.0_	70	25.0	5.0	0
F_22.5_W_7.5_	70	22.5	7.5	0
F_20.0_W_10.0_	70	20.0	10.0	0
F_17.5_W_12.5_	70	17.5	12.5	0
F_15.0_W_15.0_	70	15.0	15.0	0
F_20.0_P_7.5_W_2.5_	70	20.0	2.5	7.5
F_20.0_P_5.0_W_5.0_	70	20.0	5.0	5.0
F_20.0_P_2.5_W_7.5_	70	20.0	7.5	2.5

Notes: The total percentage of Ag fillers and WPU was set at 100 wt%, and F, P, and W represented Ag MFs, Ag NPs, and Ag NWs, respectively. F_x_, F_x_W_y_ and F_x_P_y_W_z_ implied that the Ag fillers in conductive silver pastes were single components of Ag MFs, bi-component of Ag MFs and Ag NWs, tri-component of Ag MFs, Ag NPs and Ag NWs, respectively. In addition, the subscript x, y and z indicated the percentage of Ag MFs, Ag NPs, and Ag NWs, respectively.

## Data Availability

All data used to support the findings of this study are included within the article and Supplementary Materials.

## Supplementary Materials

The following supporting information can be downloaded at: https://www.mdpi.com/article/10.3390/molecules27228030/s1, Figure S1: Schematic diagram of silver paste coating on glass substrate; Figure S2: size histogram of Ag NPs/Ag NWs/Ag MFs; Figure S3: Adhesive strength and pencil hardness test diagram of conductive silver paste paint film containing different amounts Ag MFs cured at 60 °C; Figure S4: Adhesive strength and pencil hardness test diagram of conductive silver paste sample containing different amounts of Ag MFs and Ag NPs after curing at 60 °C; Figure S5: Adhesive strength and pencil hardness test diagram of conductive silver paste sample containing different amounts Ag MFs and Ag NWs after curing at 60 °C; Figure S6: Adhesive strength and pencil hardness test diagram of conductive silver paste sample containing different amounts Ag MFs, Ag NWs and Ag NPs after curing at 60 °C; Figure S7: Pencil hardness test after conductive silver paste film formation at different curing temperatures; Figure S8: Adhesive strength test after conductive silver paste film formation at different curing temperatures; Table S1: Evaluation standard of film adhesive strength; Table S2: English Abbreviations Cross Reference List.
