# Supplementary material for: Preparation of Low Volatile Organic Compounds Silver Paste Containing Ternary Conductive Fillers and Optimization of Their Performances
Source: Molecules. 2022 Nov 19;27(22):8030. doi: 10.3390/molecules27228030 (PMC9695863; doi:10.3390/molecules27228030)
Supplement: Supplementary file 1 [file molecules-27-08030-s001.zip › molecules-2012571-supplementary.pdf]

Preparation of Low Volatile Organic Compounds Silver Paste Containing Ternary Conductive Fillers and Optimization of Their Performances

Youliang Cheng, Jin Zhang, Changqing Fang *, Wenke Qiu, Hao Chen, Haonan Liu and Ying Wei

Faculty of Printing, Packaging Engineering and Digital Media Technology, Xi'an University of Technology,

Xi'an 710048, China

* Correspondence: fangcq@xaut.edu.cn; Tel.: +86-029-61123861

Supplementary Materials

This supplement contains the following Supplementary Tables and Figures to support the manuscript:

Supplemental Table S1: Evaluation standard of film adhesive strength. (p. 2)
Table S2: English Abbreviations Cross Reference List. (p. 3)
Figure S1: Schematic diagram of silver paste coating on glass substrate. (p.3)
Figure S2: size histogram of Ag NPs/Ag NWs/Ag MFs. (p. 4)
Figure S3: Adhesive strength and pencil hardness test diagram of conductive silver paste paint film containing different amounts Ag MFs cured at 60 °C. (p. 4)
Figure S4: Adhesive strength and pencil hardness test diagram of conductive silver paste sample containing different amounts of Ag MFs and Ag NPs after curing at 60 °C. (p.5)
Figure S5: Adhesive strength and pencil hardness test diagram of conductive silver paste sample containing different amounts Ag MFs and Ag NWs after curing at 60 °C. (p. 5)
Figure S6: Adhesive strength and pencil hardness test diagram of conductive silver paste sample containing different amounts Ag MFs, Ag NWs and Ag NPs after curing at 60 ° C. (p. 6) Figure S7: Pencil hardness test after conductive silver paste film formation at different curing temperatures. (p. 6)
Figure S8: Adhesive strength test after conductive silver paste film formation at different curing temperatures. (p. 7)

Adhesive strength test of silver paste paint film: Firstly, the glass substrate coated with silver paste was placed on a hard and flat material surface, and it was cut vertically using a lattice knife with 90° cross scratches. The cutting tool should form a lattice pattern of 10×10 at specified intervals on the coating. Then, a soft bristle brush was used to remove paint chips by brushing lightly back and forth several times in the diagonal direction of the lattice array. Subsequently, the center point of the standard 75 mm transparent pressure-sensitive tape was placed on the grid, oriented parallel to a set of cutting lines, and the tape was flattened on the grid with the fingertips rubbing hard against the tape for ensuring good contact between the tape and the coating. Finally, the tape was peeled off smoothly at close 60° within 5 min. The assessment standard for adhesive strength is shown in Table S1. When the adhesive strength grade of the sample is between level 0 and 1, the 100-grid test passed.

Table S1 Evaluation standard of film adhesive strength.

Adhesive strength Grade	Results
0	The edge of the cut is completely smooth, and the edge of the lattice is free of any peeling.
1	There are small pieces of peeling at the intersection of the cuts, and the actual damage in the scribed area shall not exceed 5%.
2	The edges and/or intersections of the cuts have been peeled off, with an area of more than 5% but less than 15%.
3	There is partial peeling or large peeling along the edge of the incision, or part of the lattice is completely peeled off. The spalling area is more than 15%, but less than 35%.
4	Large scale peeling off at the edge of the incision, partial or total peeling off of some squares. The peeling area is greater than 35% but less than 65% of scribed area.
5	Exceed the previous level

Hardness test of silver paste paint film: Firstly, the sample was placed on a horizontal and stable surface. After the pencil was inserted into the pencil hardness tester and held in place, this instrument was kept horizontal. Then, the pencil load was 1.00±0.05 kg according to the national standard. Finally, when the tip of the pencil touched the surface of the paint film, the test plate was pushed to leave the direction of the operator at a speed of 0.5-1 mm/s for at least 7 mm. The hardness level was judged by the hardness of the hardest pencil that does not make a scratch of 3 mm on the coating.

Table S2 English Abbreviations Cross Reference List.

Name	Abbreviated name
Ag microflakes	Ag MFs
Ag nanowires	Ag NWs
Ag nanoparticles	Ag NPs
conductivity	C
electrical conductivity	EC
ethylene glycol	EG
electrical resistivity	ER
Poly (N-vinylpyrrolidone)	PVP
tunneling resistance	R_t
interface contact resistance	R_i
constriction resistance	R_c
volatile organic compounds	VOCs
waterborne polyurethane	WPU

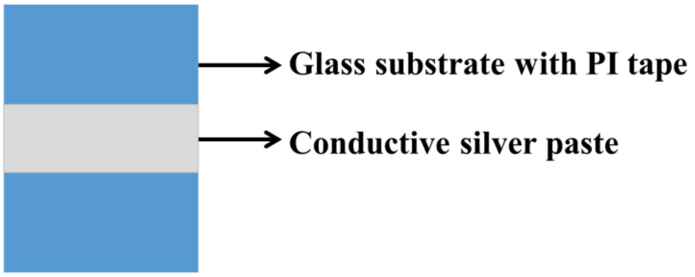

Figure S1. Schematic diagram of silver paste coating on glass substrate.

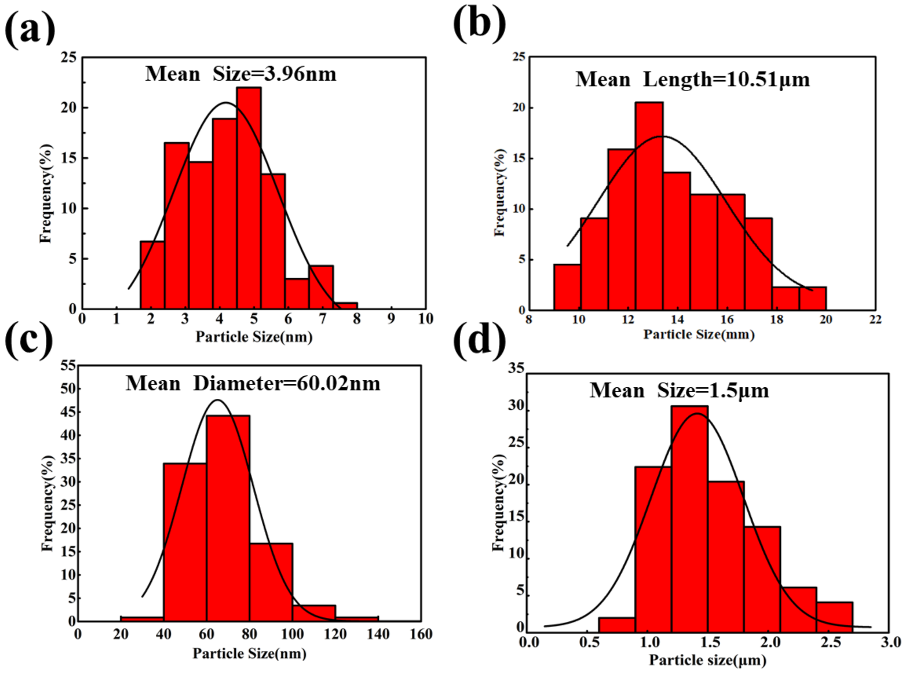

Figure S2. (a) particle size histogram of Ag NPs, (b) length histogram, and (c) diameter histogram of Ag NWs, and (d) particle size histogram of Ag MFs.

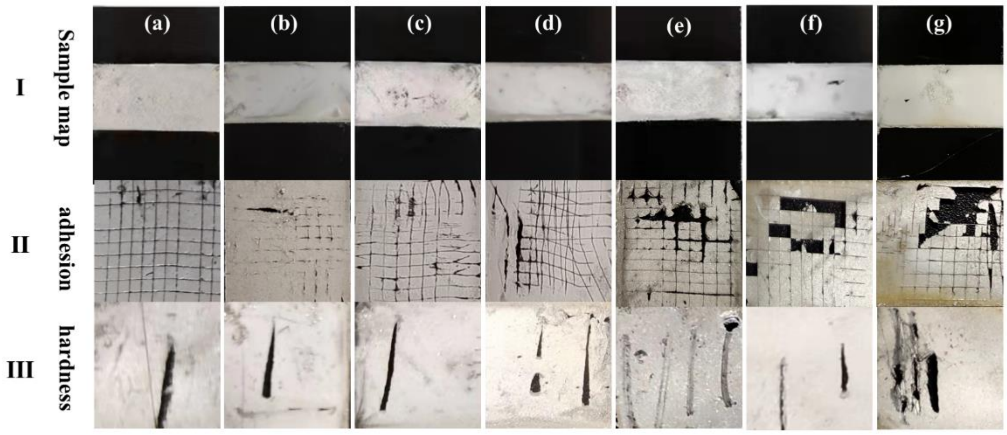

Figure S3. (I) Sample pictures of conductive silver paste paint film, (II) adhesive strength test diagram, and (III) pencil hardness test diagram of conductive silver paste paint films containing different amount Ag MFs cured at 60 °C: (a) F_{10}, (b) F_{15}, (c) F_{20}, (d) F_{25}, (e) F_{30}, (f) F_{35}, and (g) F_{40}.

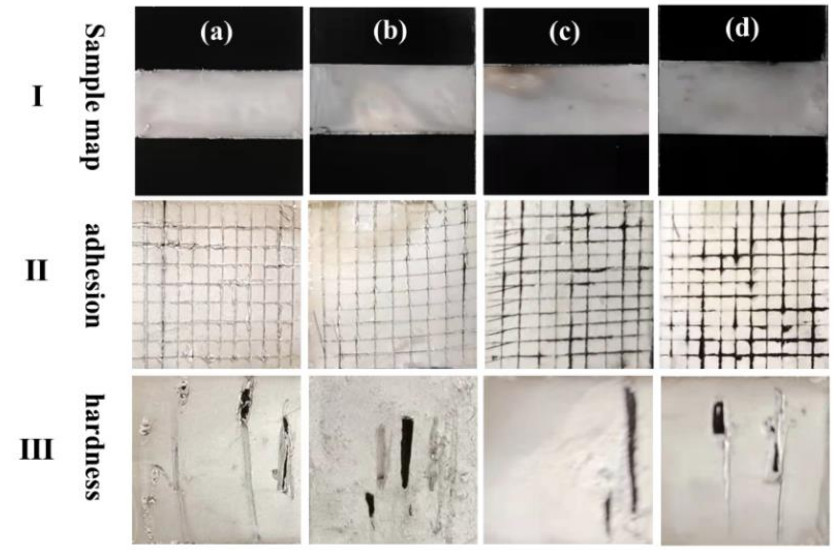

Figure S4. (I) Sample pictures of conductive silver paste paint films, (II) adhesive strength test diagrams, (III) pencil hardness test diagrams of conductive silver paste samples containing different amounts Ag MFs and Ag NPs after curing at 60 °C: (a) $F_{27.5}P_{2.5}$, (b) $F_{25.0}P_{5.0}$, (c) $F_{22.5}P_{7.5}$, and (d) $F_{20.0}P_{10.0}$.

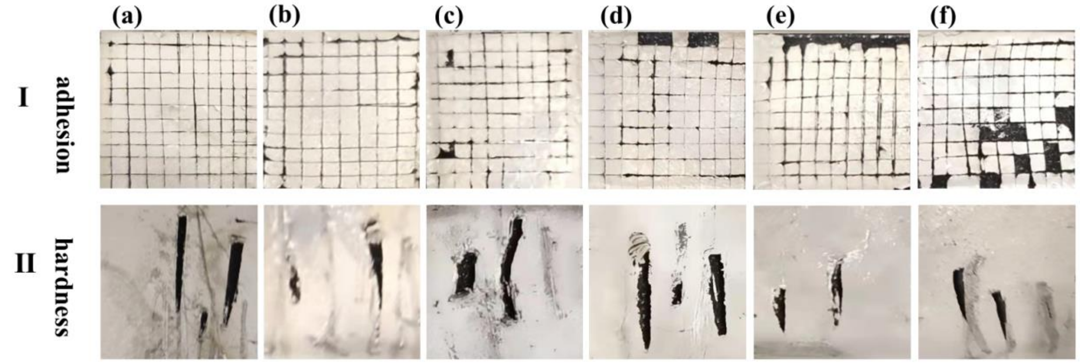

Figure S5. (I) Adhesive strength test diagram, (II) Pencil hardness test diagram of conductive silver paste sample containing different amounts Ag MFs and Ag NWs after curing at 60 °C: (a) $F_{27.5}W_{2.5}$, (b) $F_{25.0}W_{5.0}$, (c) $F_{22.5}W_{7.5}$, (d) $F_{20.0}W_{10.0}$, (e) $F_{17.5}W_{12.5}$, and (f) $F_{15.0}W_{15.0}$.

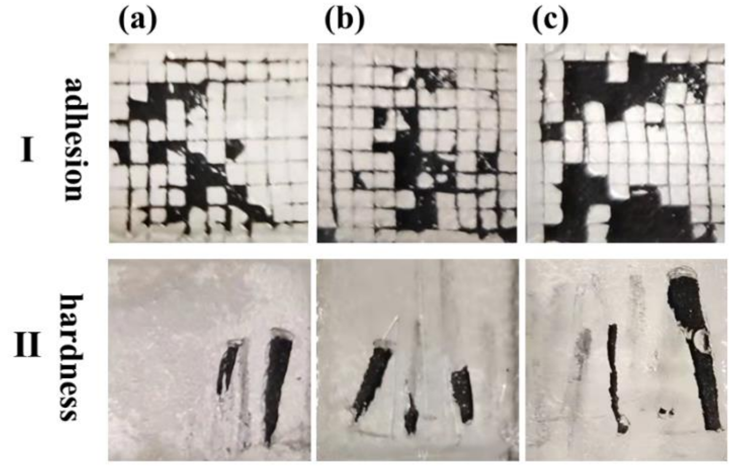

Figure S6. (I) Adhesive strength test diagram, and (II) Pencil hardness test diagram of conductive silver paste sample containing different amounts Ag MFs, Ag NWs and Ag NPs after curing at 60 °C: (a) $F_{20.0}P_{7.5}W_{2.5}$, (b) $F_{20.0}P_{5.0}W_{5.0}$, and (c) $F_{20.0}P_{2.5}W_{7.5}$.

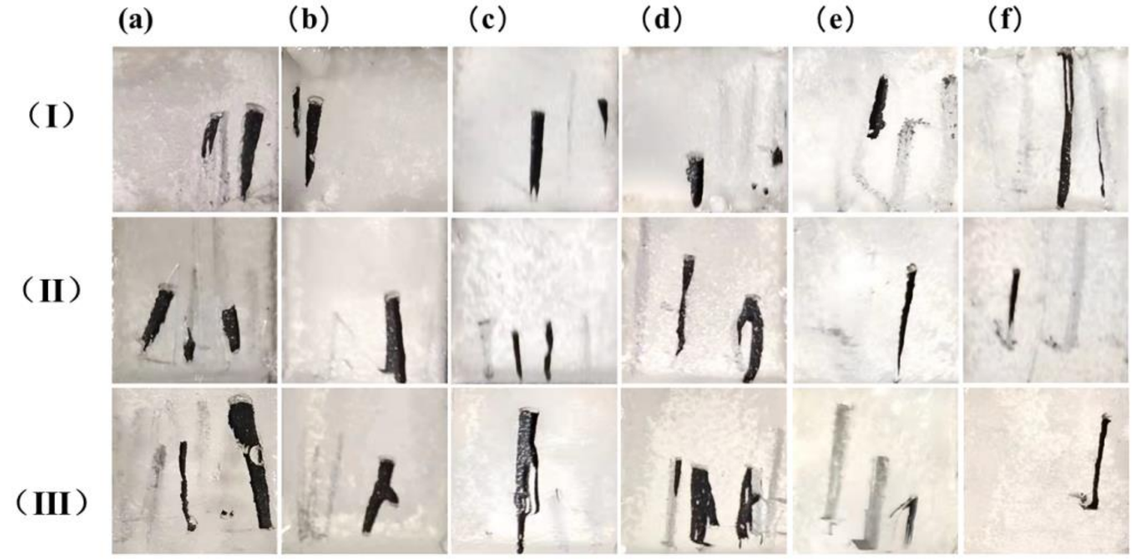

Figure S7. Pencil hardness test after conductive silver paste film formation at different curing temperatures: (a) 60 °C, (b) 80 °C, (c) 100 °C, (d) 120 °C, (e) 140 °C, and (f) 160 °C, the samples are (I) $F_{20.0}P_{7.5}W_{2.5}$, (II) $F_{20.0}P_{5.0}W_{5.0}$, and (III) $F_{20.0}P_{2.5}W_{7.5}$.

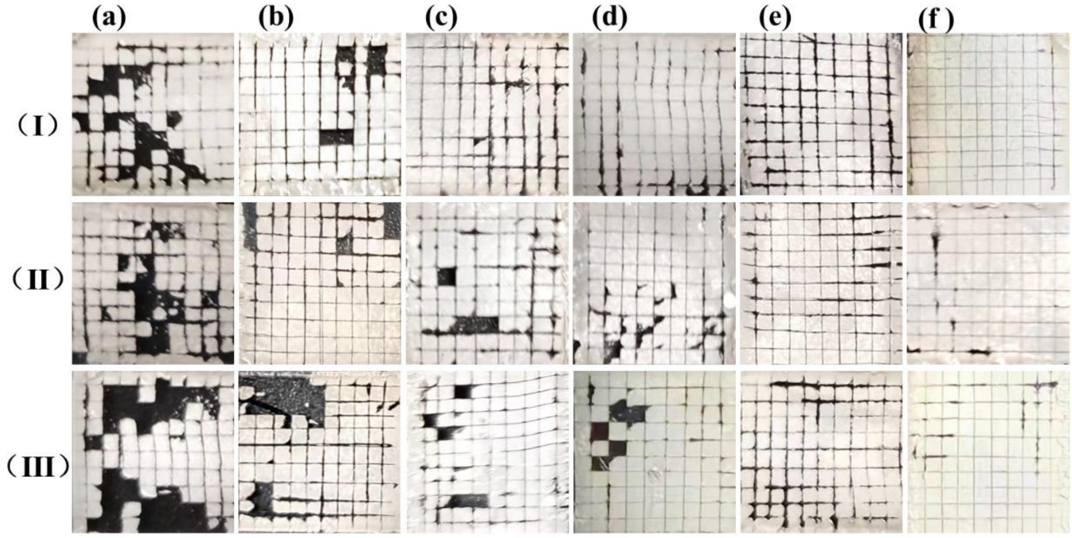

Figure S8. Adhesive strength test after conductive silver paste film formation at different curing temperatures: (a) 60 °C, (b) 80 °C, (c) 100 °C, (d) 120 °C, (e) 140 °C, and (f) 160 °C, the samples are (I) $F_{20.0}P_{7.5}W_{2.5}$, (II) $F_{20.0}P_{5.0}W_{5.0}$, and (III) $F_{20.0}P_{2.5}W_{7.5}$.
